# Effects of combined metformin and cabergoline versus metformin alone on ovarian and hormonal activities in Iraqi patients with PCOS and hyperprolactinemia: a randomized clinical trial

**DOI:** 10.25122/jml-2023-0317

**Published:** 2023-11

**Authors:** Inas Naser Hamad, Sinaa Abdul Amir Kadhim, Hayder Adnan Fawzi, Shoroq Mohammed AL-Temimi, Bassim Mohammad, Asma Swadi

**Affiliations:** 1 Department of Pharmacy, Maternity and Pediatric Teaching Hospital, Al-Qadisiyah, Iraq; 2 Department of Pharmacology, College of Medicine, University of Al-Qadisiyah, Al-Qadisiyah, Iraq; 3 Department of Pharmacy, Al-Mustafa University College, Baghdad, Iraq

**Keywords:** polycystic ovary syndrome, hyperinsulinemia, hormones, endometrial resistive index, hyperprolactinemia, PCOS: Polycystic Ovary Syndrome, T2DM: Type 2 Diabetes Mellitus, BMI: Body Mass Index, ELFA: Enzyme-Linked Fluorescent Assay, US: Ultrasound, LH: Luteinizing Hormone, FSH: Follicle-stimulating hormone, PRL: Prolactin, TSH: Thyroid-Stimulating Hormone, AMH: Anti-Müllerian Hormone

## Abstract

Polycystic ovary syndrome (PCOS) is one of the most prevalent metabolic diseases during female reproductive life, often associated with insulin resistance and hyperprolactinemia. The efficacy of metformin and cabergoline for managing PCOS remains debated in the literature. This three-arm interventional study in Iraq assessed the effects of these drugs on body mass index (BMI), hormonal balance, and uterine artery blood flow in 75 women with PCOS and hyperprolactinemia. Participants were randomized into three groups: metformin (500 mg twice daily), cabergoline (0.5 mg weekly), and a combination of both, with 25 patients in each group. Baseline and 90-day follow-up characteristics included BMI, serum hormonal levels, and ultrasound features. Metformin resulted in significant weight reduction (p=0.038); however, the addition of cabergoline caused a more significant reduction in body mass index (p=0.001). The combined treatment significantly lowered testosterone levels (p=0.008). In addition, this combination significantly reduced the level of LH (p=0.043) and increased the level of FSH (p=0.047). The results suggest that metformin and cabergoline when used together, act synergistically and safely to reduce BMI, testosterone, and LH levels while increasing FSH levels. Furthermore, this combination improved endometrial blood flow and ovulation in women with PCOS.

## INTRODUCTION

Polycystic ovary syndrome (PCOS) is commonly diagnosed in women exhibiting clinical signs such as menstrual irregularities, elevated testosterone levels, and ovarian cystic changes [1, 2]. This complex condition can manifest predominantly as hyperandrogenemia (biochemical) or be characterized by the presence of polycystic ovaries (morphological) [3]. Approximately 7% of women of reproductive age are affected by PCOS [4], making it the most common hormonal disorder in women aged 18 to 45 in the United States, where it is estimated to impact around five million individuals [5, 6]. In clinical practice, women often present with infertility, excessive hair growth, amenorrhea, acne, and obesity, leading to a diagnosis of PCOS [7]. Treatment of PCOS includes a variety of pharmacological agents. Metformin is considered an insulin sensitizer (biguanide) [8]. It does not affect insulin secretion but may enhance insulin actions [9]. It is considered the first-choice therapy for type 2 diabetes mellitus [10]. Common minor side effects include gastrointestinal intolerance [11, 12]. Metformin works by reducing blood lipid levels, decreasing hepatic glucose production, enhancing insulin-mediated glucose uptake in the liver and skeletal muscles, and reducing the use of gluconeogenic substrates [13]. Preliminary studies suggest that metformin may also improve the inflammatory state in PCOS, although the evidence remains inconclusive [14].

Cabergoline, a dopamine receptor agonist, is particularly effective on dopamine D2 receptors and has a long serum half-life of 43 hours. It is beneficial for patients with hyperprolactinemia. Studies on the use of cabergoline, a long-acting dopamine agonist, in patients with PCOS have been limited, particularly regarding its role in inhibiting prolactin (PRL) [15]. Previous reports have shown the effects of cabergoline in reducing serum pro-inflammatory cytokine levels in patients with hyperprolactinemia, but its effect on inflammation in PCOS is uncertain [16].

The lack of Iraqi literature addressing the function of metformin and cabergoline in PCOS and the inconsistent results from preliminary studies published worldwide make a clear justification for the planning and conduction of the current trial. This study aimed to explore the effects of metformin and cabergoline alone or in combination on hormonal levels and ovarian responses in a sample of Iraqi women with PCOS and hyperprolactinemia.

## MATERIAL AND METHODS

### Study design

This three-arm, parallel-group, open-label interventional study involved 75 women diagnosed with PCOS based on Rotterdam criteria (Rotterdam 2004) [17]. The diagnosis was confirmed by two specialist gynecologists after a thorough history-taking process as well as clinical examination and necessary investigations. Participants were then randomized into three groups.

1. Group M (n=25): Received oral metformin (500 mg Glucophage^®^, Merck Serono^™^) twice daily for 90 days.
2. Group D (n=25): Received cabergoline (0.5 mg Pergolin^®^, ASIA Pharmaceutical Industries) a single dose per week for 90 days.
3. Group MD (n=25): Received both metformin and cabergoline.

**Figure 1:**
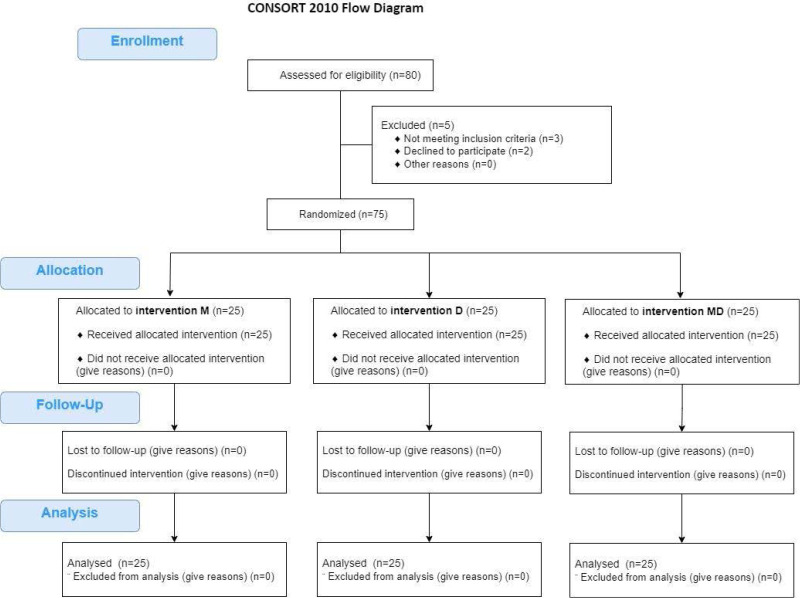
Study flowchart

Baseline and 90 days follow-up assessments included body mass index (BMI), serum hormonal levels (Anti-Müllerian Hormone (AMH), Follicle-Stimulating Hormone (FSH), Luteinizing Hormone (LH), testosterone, PRL, Triiodothyronine (T3), Thyroxine (T4), and Thyroid-Stimulating Hormone (TSH)), and ultrasonic features (number and size of dominant follicles and uterine artery resistive index). The study flow is illustrated in Figure 1 and was conducted in accordance with the CONSORT 2010 statement.

### Study setting and participants

Participants were recruited from the gynecology outpatient clinic of the Maternity and Pediatrics Teaching Hospital in Al-Diwaniyah Province, Iraq, between 21 September 2022, and 31 March 2023. Laboratory tests and ultrasound examinations were also conducted at this facility. Eligible participants were female patients aged 18-40, diagnosed with PCOS based on the Rotterdam 2004 criteria [17], with a BMI less than 40 kg/m^2^ and PCOS with hyperprolactinemia. Patients with co-morbid conditions such as diabetes mellitus, essential hypertension, and thyroid disease, as well as those planning for conception, were excluded from the study.

### Randomization and sample size estimation

Participants were randomized using a computer-based system (Research Randomizer) following PCOS diagnosis. Sample size estimation was based on the following equation:

Minimal sample size (n) = pX(1-p)XZ^2^/Me^2^

The sample size was calculated using the formula: n = pX(1-p)XZ^2^/Me^2^, where 'p' was the prevalence of PCOS (20% as per Deswal *et al*. [18]), 'Z' was the Z-score at a 95% confidence interval (1.96), and 'Me' was the marginal error (0.05 as per another study) [19]. Subsequently, the minimal sample size was estimated to be approximately 24.6 and accordingly rounded up to 25 for each group. This study was registered with the Clinical Trials Registry (Identifier: NCT05981742).

### Procedures and measurements

BMI was calculated using the standard formula: BMI = weight in kg/height in m^2^ [20]. Ultrasound examinations were performed using a grayscale and colored Doppler US (Voluson™ E6 GE HealthCare Technologies) with a 3–5 MHz curved surface transducer. Dominant follicles were counted manually using real-time 2D-US, and the mean uterine artery resistive index was calculated via Doppler study.

### Laboratory sample preparation and analysis

A 10 ml venous blood sample was collected from each patient. After coagulation, the blood was centrifuged at 2,000 to 3,000 rpm for 20 minutes. Hormonal levels were measured using an automated enzyme-linked fluorescent assay (ELFA) to determine the required material concentration in human serum (MINI VIDAS^®^ bioMérieux). The specific hormones measured and their respective kits were as follows:

- Follicular-Stimulating Hormone (FSH): Measured using the VIDAS^®^ FSH kit (bioMerieux, Ref. no.: 30 407-01).
- Luteinizing Hormone (LH): Measured using the VIDAS^®^ LH kit (bioMerieux, Ref. no.: 30 406-01).
- Testosterone: Measured using the VIDAS^®^ Testosterone II kit (bioMerieux, Ref. no.: 414320).
- Prolactin (PRL): Measured using the VIDAS^®^ Prolactin kit (bioMerieux, Ref. no.: 30 410-01).
- Triiodothyronine (T3): Measured using the VIDAS^®^ T3 kit (bioMerieux, Ref. no.: 30 403-01).
- Thyroxine (T4): Measured using the VIDAS^®^ T4 kit (bioMerieux, Ref. no.: 30 404-01).
- Thyroid-Stimulating Hormone (TSH): Measured using the VIDAS^®^ TSH kit (bioMerieux, Ref. no.: 30 400-01).
- Anti-Mullerian Hormone (AMH): Measured using the VIDAS^®^ AMH kit (bioMerieux, Ref. no.: 417011).

### Statistical analysis

One-way analysis of variance (ANOVA) was applied to normally distributed variables, while the Kruskal-Wallis test was used for non-normally distributed variables. Paired t-tests were conducted to compare pre-and post-treatment means within each group. The level of significance was set at a P-value of 0.05 or less.

## RESULTS

### Demographic, laboratory, and hormonal characteristics

There was no statistically significant difference in the mean age across the study groups (p=0.149), with ages ranging between 15 and 39 years (Table 1). The mean age across groups was 24.80±4.87 years, 24.24±4.54 years, and 22.32±4.54 years, respectively, for MD, M, and D groups. In addition, there were no significant differences in the mean BMI among study groups (p=0.231). The mean BMI across study groups were 29.09±4.49 kg/m^2^ (group MD), 30.18±4.55 kg/m^2^ (group M), and 29.21±2.92 kg/m^2^ (group D), respectively (Table 1).

**Table 1:** Comparison of demographic, laboratory, and hormonal parameters among study groups

Parameters	Group MD	Group M	Group D
BMI (kg/m^2^)			
Baseline	29.09±4.49	30.18±4.55	29.21±2.92
After 90 days	27.31±3.84	29.00±4.73	28.68±2.16
p-value	0.001	0.038	0.183
AMH (ng/mL)			
Baseline	6.35±2.40	5.48±2.77	6.42±2.34
After 90 days	5.57±2.74	4.59±2.38	5.63±2.09
p-value	0.216	0.056	0.117
Testosterone (ng/mL)			
Baseline	0.49±0.17	0.43±0.20	0.44±0.17
After 90 days	0.37±0.13	0.68±0.95	0.49±0.27
p-value	0.008	0.221	0.533
LH (mIU/mL)			
Baseline	7.19±2.32	7.54±3.02	7.25±2.48
After 90 days	5.81±2.68	6.25±2.83	6.09±2.45
p-value	0.043	0.078	0.092
FSH (mIU/mL)			
Baseline	7.01±2.56	7.30±3.01	7.10±1.01
After 90 days	8.58±2.24	6.57±2.54	7.19±0.79
p-value	0.047	0.257	0.261
Prolactin ng/mL			
Baseline	17.26±6.66	15.82±9.43	24.98±32.45
After 90 days	1.79±1.47	16.08±14.89	4.92±10.12
p-value	<0.001	0.491	0.004

Data presented as mean ± SD.

SD: standard deviation; BMI: body mass index; AMH: Anti-Müllerian hormone; LH: luteinizing hormone; FSH: follicle stimulating hormone.

Metformin alone resulted in significant weight reduction, but cabergoline alone did not affect body weight significantly. However, when combined, metformin and cabergoline induced a more substantial decrease in BMI. Regarding hormonal changes, neither metformin nor cabergoline alone significantly altered serum testosterone levels, but their combination resulted in a significant reduction (p=0.008). Both drugs, in combination, significantly decreased the level of LH and increased the level of FSH (p<0.05), but the use of either drug alone failed to do so. Regarding serum PRL, metformin alone did not lead to a statistically significant reduction. However, cabergoline effectively reduced PRL levels (p=0.004), and the combination of both drugs resulted in an even more significant reduction (p<0.001).

### Changes in ultrasound characteristics

Changes in the mean number of dominant follicles and mean resistive index are shown in Table 2. In women with PCOS, treatment with metformin alone significantly increased the number of dominant follicles, and this increase was even more pronounced when both drugs were used together. In contrast, cabergoline alone did not significantly affect the number of dominant follicles. Additionally, the resistive index decreased following the use of metformin alone and with the combination of both drugs, whereas cabergoline alone did not produce a significant change in the resistive index.

**Table 2:** Comparison of ultrasonic parameters

Parameters	Group MD	Group M	Group 3 D
Dominant Follicles (DF)			
Baseline	12.72±3.96	12.00±3.79	13.14±3.83
After 90 days	19.08±3.37	14.92±3.19	13.16±3.26
p-value	<0.001	<0.004	0.381
Resistive index (RI)			
Baseline	0.88±0.08	0.91±0.08	0.89±0.10
After 90 days	0.71±0.09	0.78±0.09	0.87±0.09
p-value	<0.001	<0.001	0.181

Data presented as mean ± SD.

SD: standard deviation, DF: number of dominant follicles, RI: resistive index.

## DISCUSSION

In the present study, we observed that metformin alone led to a significant weight reduction among women with PCOS, which was even more pronounced after the addition of cabergoline. Nevertheless, cabergoline alone did not affect body weight significantly. This finding aligns with existing research indicating the effectiveness of metformin in reducing weight in patients with PCOS [21, 22]. Additionally, some studies have suggested that cabergoline can reduce body weight in women with PCOS [23].

Ghaneei *et al*. compared the effect of metformin alone and in combination with cabergoline on 110 women with PCOS. They reported a similar significant weight reduction with both treatments but did not observe any additional benefit from adding cabergoline [15]. Similarly, Elsersy's study involving 250 patients with PCOS with elevated serum PRL levels also aligns with our observations. In this study, one group received metformin and cabergoline, while the control group received metformin and a placebo. The results showed significant weight reduction with metformin alone and an even greater reduction with the addition of cabergoline. However, they had no group of cabergoline alone to compare with [24].

Our findings are consistent with those reported by Pala *et al*. [25], who observed that normalizing PRL levels using dopamine agonists can reverse the abnormalities in body composition and metabolism caused by hyperprolactinemia. The role of cabergoline, a dopamine agonist, in reducing body mass index can be understood through its multifaceted effects on dopaminergic transmission. Dopamine not only influences PRL secretion but also plays a crucial role in regulating eating behavior, insulin sensitivity, and energy distribution [25]. Studies have shown that in obese rats, as well as in humans, dopamine levels and/or function are often decreased [26-29]. This reduction impacts dopaminergic reward pathways that originate in the mesencephalic nuclei and then sends the axons to the specific areas of the brain involved in the motivational (striatum), emotional (limbic system), and higher-order functions (frontal cortex). There are five types of receptors divided into two major classes: D1R-like (D1 and D5) and D2R-like (D2, D3, and D4) receptors. Functional evaluation of components of eating behavior suggests that D1 agonists reduce meal frequency in rodents, but D2 agonists reduce the quantity of meals [26]. Neuroimaging studies have shown that individuals with obesity tend to have significantly lower levels of D2/D3 receptors [27]. This reduction may lead to decreased sensitivity to reward stimuli, potentially resulting in increased food-seeking behavior. Furthermore, antipsychotic medications that inhibit D2 receptors are associated with heightened appetite and metabolic issues. These issues include weight gain, exacerbation of pre-existing diabetic conditions, and in some cases, the onset of new diabetes even without weight gain [28, 29]. In contrast, pharmacological agents that increase dopamine release or block the reuptake can decrease animal and human food consumption [30].

Extensive cohort studies have established a link between metformin use and weight reduction benefits. Traditionally, these effects were attributed to metabolic mechanisms, such as decreased hepatic glucose production and insulin secretion. Emerging evidence demonstrates that hypothalamic appetite control centers are modulated by metformin, resulting in weight reduction, as well as alterations in gastrointestinal (GIT) microbiota and the reversing aging effects. Metformin is also being studied for the treatment of problems connected to obesity, including hepatic steatosis, sleep apnea, and osteoarthritis [31].

In the present study, we observed no significant change in serum levels of AMH in all groups, indicating that the administration of metformin and/or cabergoline did not have a significant impact on serum AMH levels in patients with PCOS. This finding aligns with the research by Grigoryan *et al*. who also reported no significant impact on serum AMH levels after six months of metformin treatment (850 mg twice daily) [32]. However, this contrasts with the findings of Rahmanian *et al*. [33], who observed a significant reduction in AMH levels among patients with PCOS treated with metformin. Similarly, Foroozanfard *et al*. supported the results of Rahmanian *et al*. [34]. Numerous studies have demonstrated that metformin regulates menstrual periods and improves ovulation in patients with PCOS. However, the effect of metformin on blood AMH levels in these individuals remains unclear [35].

Furthermore, we found that metformin significantly reduced testosterone levels in women with PCOS, whereas cabergoline did not have this effect. These findings are in line with the research conducted by Kazerooni *et al*., who showed that metformin treatment reduced testosterone levels in individuals with PCOS and significantly improved the clinical manifestations of hyperandrogenism [36]. In the 1990s, it was discovered that metformin decreased testosterone levels in women with PCOS, probably as a result of its insulin-sensitizing effects. However, the specific actions behind the effects of metformin on PCOS remain not clearly known, and there is substantial variation in the clinical response to this medication among women with PCOS [36].

In this study, metformin significantly increased LH and FSH levels in women with PCOS, but cabergoline failed to do so. However, these findings contrast with the meta-analysis by Guan *et al*. which reported an overall significant reduction in FSH and LH levels following metformin administration [37]. Similarly, Curi *et al*. observed no significant change in FSH and LH levels after metformin treatment in women with PCOS [38]. These discrepancies highlight the controversial nature of FSH and LH changes in response to metformin, underscoring the need for further research to reach a consensus on these hormonal responses. Furthermore, we observed that metformin did not significantly reduce PRL levels in women with PCOS, but cabergoline was effective in doing so. This was expected because cabergoline is a dopamine antagonist, and its main clinical use is to treat hyperprolactinemia.

Another notable finding was that metformin alone significantly increased the number of dominant follicles in patients with PCOS, whereas cabergoline alone did not have this effect. Theoretically, metformin and other insulin sensitizers reduce hyperandrogenism and hyperinsulinemia in patients with PCOS and revert the ovulatory cycle to normal [39]. Several studies have documented the positive benefits of adding metformin to the treatment regimen of patients with PCOS who are clomiphene citrate-resistant. This combination has been shown to significantly enhance ovulation rate and improve most outcome measures [40, 41]. However, other studies shown that the addition of metformin enhanced the ovulation rates [42, 43]. Because metformin is significantly more affordable than gonadotropins, it is recommended to initiate ovulation induction with metformin before starting therapy with gonadotropins.

We observed a reduction in the resistive index following metformin alone, while cabergoline alone did not produce this effect. Previous research has demonstrated improvements in endometrial vascularity following the administration of metformin in patients with PCOS, as reported by several authors [44, 45]. Orio *et al*. found that endothelin 1 (ET-1), a vasoconstrictive peptide produced in the endothelial cells of blood vessels, is elevated in women with PCOS. Furthermore, metformin treatment was shown to enhance this change [46]. The positive impact of metformin on ET-1 levels may represent an indirect mechanism through which this medication affects uterine vascularity. ET-1 is expressed in the endometrium of humans and is inversely related to the plasma levels of estradiol [47], indicating that they both might work together, regulating blood flow to the endometrium. Metformin may influence uterine perfusion by decreasing androgen levels [48, 49], exerting a vasoconstrictive impact on vascular tissues [50]. This study had several limitations. Firstly, the short duration of the study may not have been sufficient to fully assess the long-term effects of the treatments. Secondly, the open-label nature of the study could introduce potential biases, as opposed to the more controlled conditions of double-blinded trials.

## CONCLUSION

Metformin and cabergoline were found to be effective and safe when used in combination, acting synergistically to reduce body mass index and testosterone levels and to lower LH levels in women with PCOS and hyperprolactinemia. Additionally, this combination increased serum FSH levels and improved endometrial blood flow and ovulation in these patients.

## Data Availability

Further data is available from the corresponding author on reasonable request.
